# Cancer immunotherapy: weak beats strong

**DOI:** 10.18632/aging.101134

**Published:** 2016-11-30

**Authors:** Wolfgang Deppert, Michael Bruns

**Affiliations:** Heinrich-Pette-Institute, Leibniz-Institute for Experimental Virology, 20251 Hamburg, Germany

**Keywords:** immune checkpoint blockade therapy, tumor antigen T-cell epitopes, tumor regression

Immune checkpoint blockade therapy, i.e. the ablation of control by elimination of checkpoint regulating proteins is a promising novel tool in cancer therapy. Such therapies are designed to restore the patients' own antitumor immune response, specifically the activity of cytotoxic T-cells (CTLs), which had been mitigated during the processes of tumor immune evasion. Immune checkpoint blockade can be induced by treatment with appropriate antibodies against checkpoint proteins, like e.g. antibodies against programmed death-1 protein (PD1) or its ligand (PD-L1) that disrupt the PD1/PD-L1 immune checkpoint axis [1]. Although such approaches are promising, they are successful only in a limited fraction of patients. Unfortunately, factors that influence therapy response in such approaches are not well understood so far. It has been reported that tumors that express many neo-antigens due to accumulation of frame shift or point mutations are more susceptible to immune checkpoint blockade than tumors lacking this property [2], suggesting that immunogenicity of tumor antigen T-cell epitopes might play an important role for therapy success or failure. For obvious reasons functional studies supporting this conclusion are difficult to perform in humans, and thus require the use of suitable animal models.

Using the well characterized and cross-species validated BALB/c mouse based WAP-T models for triple-negative breast cancer [3,4], we assessed the role of tumor antigen T-cell immunogenicity in PD1/PD-L1 immune checkpoint blockade therapy [5]. We compared the response to anti-PD1/PD-L1 antibody therapy in two different lines of tumor mice (WAP-T and WAP-T_NP_ mice, respectively) immunologically differing only in the expression of a single T-cell epitope in their major tumor antigen: WAP-T and WAP-T_NP_ mice contain both as transgene the SV40 early gene region under control of the whey acidic protein (WAP) promoter, which upon induction codes for SV40 early proteins, with T-antigen being the major tumor antigen. In WAP-T_NP_ mice, the SV40 transgene additionally encodes a highly immunogenic T-cell epitope, the NP_118-126_-epitope within the nucleoprotein (NP) of lymphocytic choriomeningitis virus (LCMV). While SV40 T-antigen (T-Ag) expressed in WAP-T tumor mice is only weakly immunogenic in the BALB/c mouse background, the chimeric T-Ag/NP protein (T-Ag_NP_) in WAP-T_NP_ tumor mice is highly immunogenic. Except for this immunological difference, WAP-T and WAP-T_NP_ tumors are histologically and molecularly extremely similar [6]. We asked, whether in comparison to the weakly immunogenic T-cell epitopes of T-Ag in WAP-T tumor mice the presence of the highly immunogenic NP-epitope in T-Ag_NP_ influences anti-PD1/PD-L1 antibody therapy response. The expectation was that reactivation of the potent NP-epitope specific CTLs in WAP-T_NP_ tumor mice would provide better protection against tumor re-growth than reactivation of the significantly less potent T-Ag-specific CTLs.

**Figure 1:**
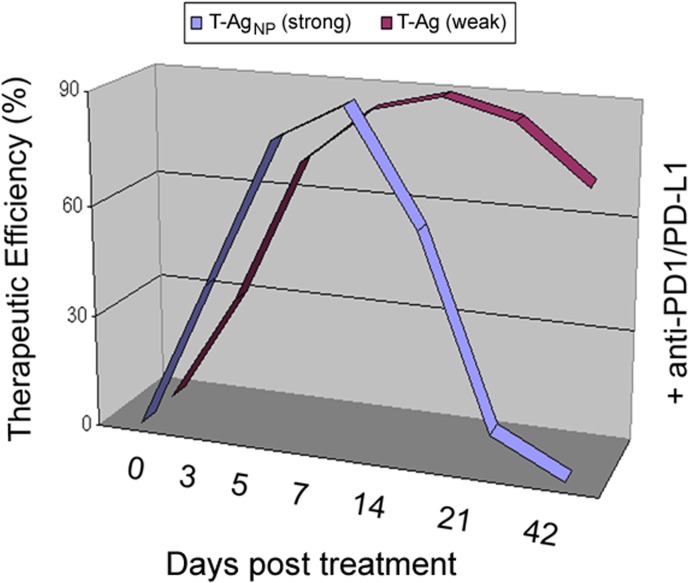
Response of WAP-T tumor mice, presenting weak CTL epitopes in T-Ag, and of WAP-T_NP_ tumor mice, additionally presenting the strong LCMV NP-epitope in T-Ag_NP_, to treatment with antibodies directed against PD1 or PD-L1 proteins. The period of tumor regression is significantly extended in WAP-T mice.

Treatment of WAP-T_NP_ tumor mice with either anti-PD1 or anti-PD-L1 antibodies led to almost complete tumor regression. However, tumors began to reappear already less than two weeks after treatment and had fully reached their pre-treatment size after 21 days, indicating that CTL exhaustion had been rapidly re-established. Surprisingly, the same treatment applied to WAP-T tumor mice resulted in a significantly prolonged period of tumor regression (up to 31 days compared to less than 14 days in WAP-T_NP_ tumor mice (Fig. 1). Due to the close similarities of WAP-T and WAP-T_NP_ tumors, this difference can only be ascribed to the presence or absence of the highly immunogenic NP-epitope in WAP-T and WAP-T_NP_ tumors, respectively. Further experiments provided evidence that the strong immunogenicity of the NP-epitope in T-Ag_NP_ indeed elicited a fast and strong epitope-specific CTL response, but at the same time also promoted rapid CD8^+^ T-cell exhaustion. Thus during and after treatment, residual WAP-T_NP_ tumor cells will induce new active NP-specific CTLs, which together with residual non-exhausted CTLs will kill most of the tumor cells. These CTLs, however, will rapidly become exhausted in the tumor-supporting microenvironment, thereby allowing tumor re-growth. On the other hand, the relatively good efficacy of the anti-PD1/PD-L1 treatment in WAP-T tumor mice supports the idea that tumors expressing weak tumor antigen T-cell epitopes respond much better to immune checkpoint blockade therapies because re-establishment of an exhausted status of CTLs against these epitopes takes much longer. The data support the view that immunogenicity of tumor antigen T-cell epitopes strongly influences the duration of an anti-PD1/PD-L1 induced immune checkpoint blockade, and thus is an important parameter in determining the outcome of an immune checkpoint blockade therapy.

The findings open new avenues for improving the success of immune checkpoint blockade therapies: first, methods for assessing relative T-cell epitope strengths in different HLA subtypes are progressing [7], which will allow selecting patients amenable to immune checkpoint blockade therapy; second, it should be possible to analyze, why weak tumor antigen T-cell epitopes favor and strong ones prevent a prolonged activity of CTLs after their reactivation by immune therapy. Understanding the respective pathways at the molecular level will allow identification of targets for improving such therapies.
